# P-639. Predictive Model to Identify Adult Patients with RSV at Risk of Developing Severe Acute Respiratory Infection

**DOI:** 10.1093/ofid/ofaf695.852

**Published:** 2026-01-11

**Authors:** Jose A Castro Cordero, Juan Villalobos Vindas

**Affiliations:** Caja Costarricense de Seguro Social, Uruca, San Jose, Costa Rica; Caja Costarricense de Seguro Social, Uruca, San Jose, Costa Rica

## Abstract

**Background:**

Respiratory Syncytial Virus (RSV) infection in adults can evolve into severe conditions. Identifying risk factors for Severe Acute Respiratory Infection (SARI) would optimize clinical management. To develop and validate a predictive model to identify adult patients with RSV at risk of developing SARI.

**Figure:**
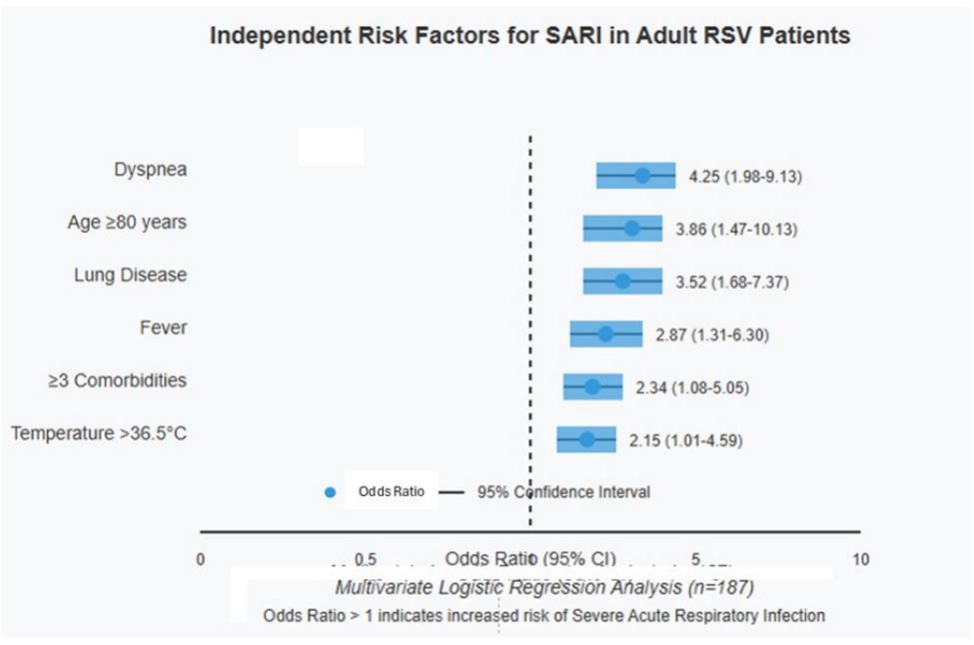
Independent Risk Factors Independent Risk Factors for SARI in Adult RSV Patients

**Figure:**
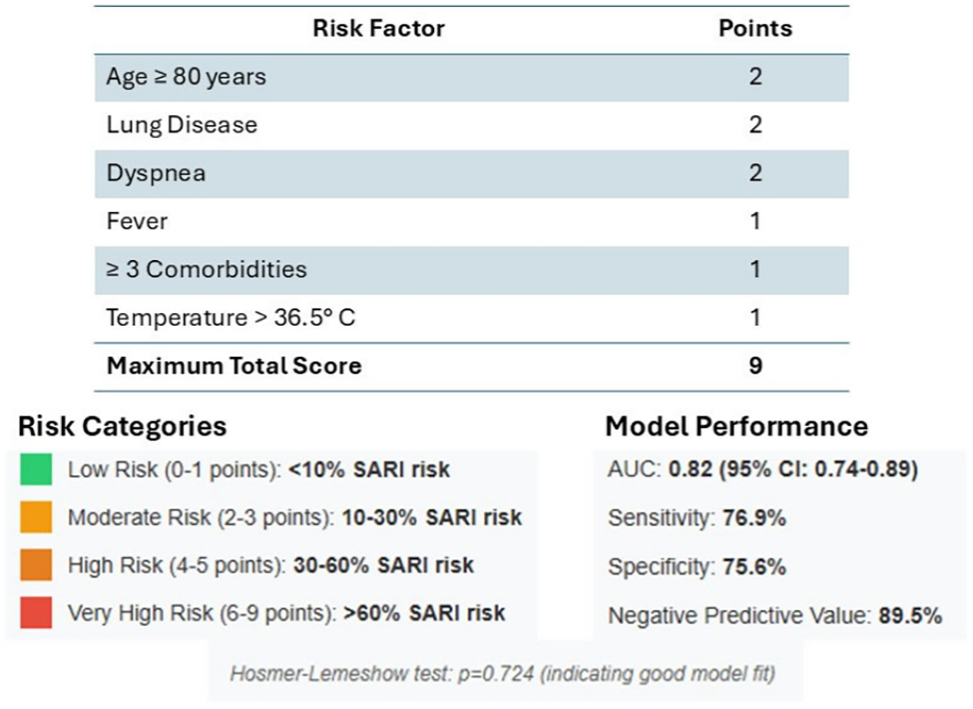
Clinical Prediction Score Clinical Prediction Score for SARI Risk in Adult RSV Patients

**Methods:**

Analysis of 187 adults with PCR-confirmed RSV (Mexico Hospital, 2022-2024). Using multivariate logistic regression, independent factors associated with SARI were identified. A clinical scoring system was developed, and its diagnostic performance was evaluated through ROC analysis and internal validation by bootstrap.

**Figure:**
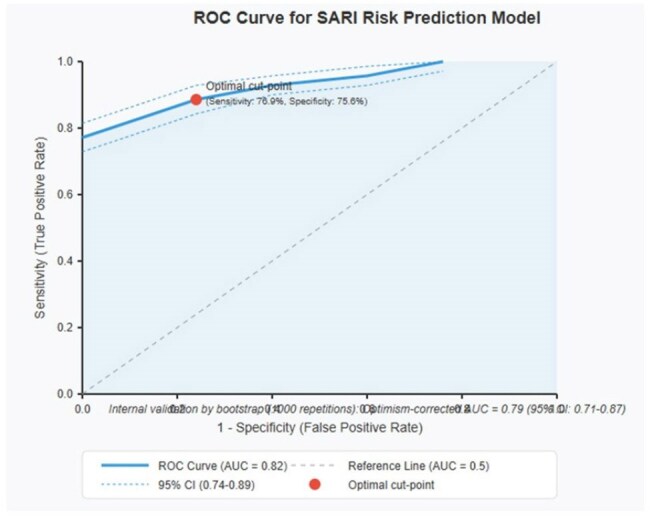
ROC Curve ROC Curve for SARI Risk Prediction Model

**Figure:**
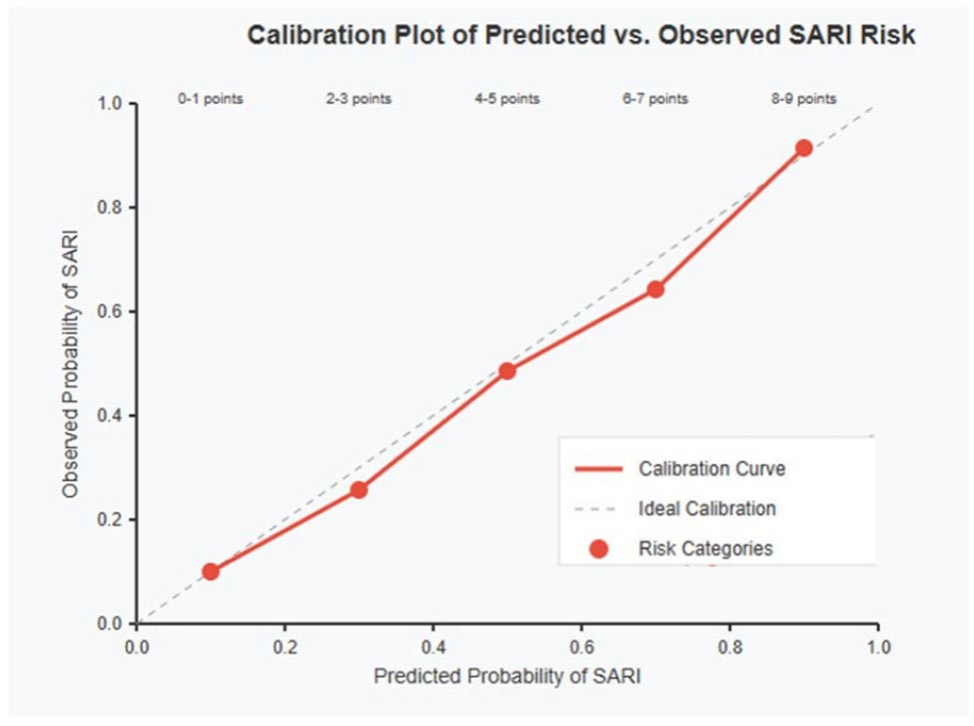
Calibration Plot Calibration Plot of Predicted vs Observed SARI Risk

**Results:**

Six independent risk factors for SARI were identified: age ≥80 years (OR 3.86, 95%CI 1.47-10.13), preexisting lung disease (OR 3.52, 95%CI 1.68-7.37), presence of dyspnea (OR 4.25, 95%CI 1.98-9.13), fever (OR 2.87, 95%CI 1.31-6.30), ≥3 comorbidities (OR 2.34, 95%CI 1.08-5.05), and temperature >36.5°C (OR 2.15, 95%CI 1.01-4.59). The scoring system assigned 2 points to age ≥80 years, lung disease, and dyspnea; 1 point to fever, ≥3 comorbidities, and temperature >36.5°C. The model showed excellent discriminative capacity (AUC 0.82, 95%CI 0.74-0.89) with sensitivity 76.9%, specificity 75.6%, and negative predictive value 89.5%. Internal validation confirmed the model's robustness (optimism-corrected AUC 0.79).

**Conclusion:**

The developed scoring system effectively stratifies SARI risk in adult patients with RSV, with four categories: low risk (0-1 points, < 10%), moderate (2-3 points, 10-30%), high (4-5 points, 30-60%), and very high (6-9 points, >60%). This tool could optimize clinical decisions, facilitating identification of patients requiring closer monitoring or early intervention. External validation is required before widespread implementation.

**Disclosures:**

All Authors: No reported disclosures

